# Rubinstein-Taybi syndrome: clinical features, genetic basis, diagnosis, and management

**DOI:** 10.1186/s13052-015-0110-1

**Published:** 2015-01-20

**Authors:** Donatella Milani, Francesca Maria Paola Manzoni, Lidia Pezzani, Paola Ajmone, Cristina Gervasini, Francesca Menni, Susanna Esposito

**Affiliations:** Pediatric Highly Intensive Care Unit, Department of Pathophysiology and Transplantation, Università degli Studi di Milano, Fondazione IRCCS Ca’ Granda Ospedale Maggiore Policlinico, Via Commenda 9, 20122 Milano, Italy; UO Neuropsichiatria dell’Infanzia e dell’Adolescenza, Fondazione IRCCS Ca’ Granda Ospedale Maggiore Policlinico, Milano, Italy; Department of Health Science, Medical Genetics, Università degli Studi di Milano, Milano, Italy

**Keywords:** *CREBBP*, Intellectual disability, Plurimalformative syndrome, Rubinstein syndrome, Rubinstein-Taybi syndrome

## Abstract

**Background:**

Rubinstein-Taybi syndrome (RSTS) is an extremely rare autosomal dominant genetic disease, with an estimated prevalence of one case per 125,000 live births. RSTS is characterized by typical facial features, microcephaly, broad thumbs and first toes, intellectual disability, and postnatal growth retardation. However, no standard diagnostic criteria are available for RSTS. In this review, we summarized the clinical features and genetic basis of RSTS and highlighted areas for future studies on an appropriate diagnostic protocol and follow-up care for RSTS.

**Discussion:**

RSTS is primarily characterized by delayed growth in height and weight, microcephaly, dysmorphic facial features, and broad thumbs and big toe. Over 90% RSTS individuals with disabilities survive to adulthood, but healthcare for these patients is particularly complex, time-consuming, and costly. In addition, no standard diagnostic criteria and follow-up care guidelines are available for RSTS. It has been shown that mutations in the genes encoding the cyclic-AMP-regulated enhancer binding protein (*CREBBP*) and the E1A-binding protein p300 (*EP300*) contributed to the development of RSTS. Therefore, genetic tests are useful for the diagnosis of RSTS, although most RSTS cases are currently diagnosed based on clinical features.

**Summary:**

The clinical features of RSTS have been extensively studied, which significantly contributes to the diagnosis of this extremely rare syndrome. However, the pathogenesis and genotype-phenotype associations of RSTS are largely unknown. Therefore, multicenter studies and international cooperation are highlighted for better understanding of this disease, establishing standard diagnostic criteria, and providing professional management and follow-up care of RSTS.

## Background

Plurimalformative syndromes, which are named according to their low prevalence and incidence in the population, consist of a large group of rare diseases. Rubinstein-Taybi syndrome (RSTS, OMIM #180849, #613684) is an extremely rare disease and was first described in 1963 [1]. The incidence of RSTS is 1 in 100,000 to 125,000 live births. Currently, no precise diagnostic criteria are available, although RSTS is primarily characterized by poor postnatal height-weight growth, intellectual disability, microcephaly, dysmorphic facial features, broad thumbs, and big first toes. While a number of major malformations and clinical complications are associated with RSTS, these signs and symptoms cannot be considered pathognomonic to RSTS. Until the 90s, diagnosis has remained exclusively clinical and radiological (x-ray of hands and feet). The genetic bases were first identified in 1991, demonstrating a *de novo* reciprocal translocation with breakpoints in chromosomal region 16p13.3 in some patients [2-4]. Subsequently, affected subjects were analyzed using Fluorescent In Situ Hybridization (FISH). In six cases, the hybridization signal was present on only one allele on 16p13.3, confirming that the absence of this region lead to RSTS [5,6]. Additional research has led to the discovery of mutations in the gene encoding cyclic-AMP-regulated enhancer binding protein (*CREBBP*) in 16p13.3 in RSTS patients [7]. Mutations of *CREBBP* gene were reported in approximately half of RSTS patients [8,9]. *CREBBP* gene and its homolog, E1A binding protein p300 (*EP300*) on chromosome 22, are involved in a number of basic cellular activities, such as DNA repair, growth, differentiation, apoptosis of cells, and tumor suppression by serving as transcriptional co-activators in different signaling pathways [10]. As *CREBBP* and *EP300* interact closely, studies have been conducted to investigate whether mutations in *EP300* were associated with RSTS. The results of *EP300* sequencing in a group of six subjects revealed three mutations [11]. Subsequently, the incidence of *EP300* mutations was estimated as about 5-8% [12-16]. Generally, 55-70% clinically diagnosed RSTS cases were confirmed through genetic testing [17]. In this review, we discussed the clinical features and genetic studies of RSTS and we try to outline future directions for an appropriate clinical diagnosis and follow-up of this condition.

## Discussion

### Typical features

**Figure 1:**
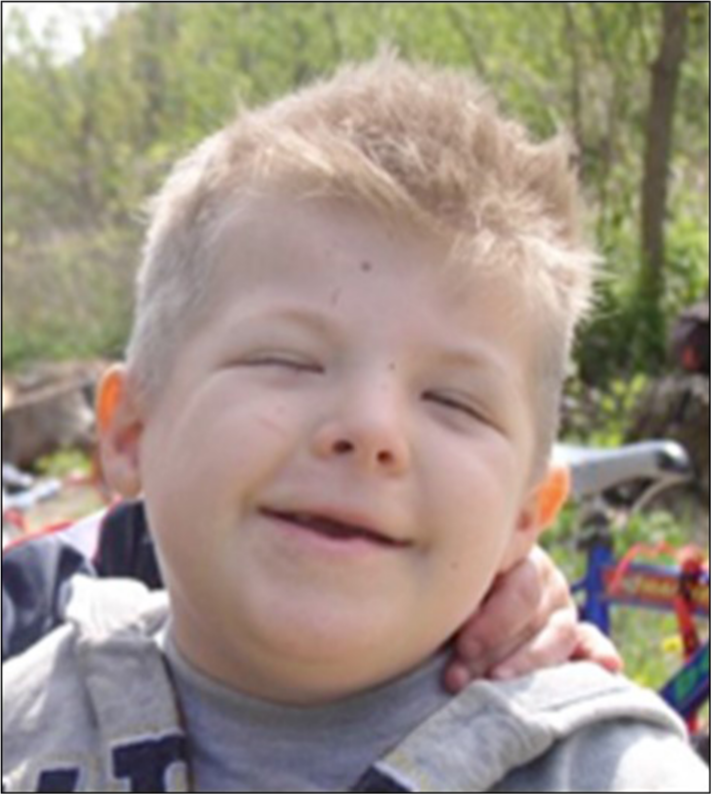
**Typical facies of a RSTS patient, including arched eyebrows, slanted palpebral fissures, protruding beaked nose with columella below alae nasi, arched palate, mild micrognathia, labial commissures facing upward, teeth anomalies, and an atypical smile (“grimacing”) with nearly completely closed eyes.**

**Figure 2:**
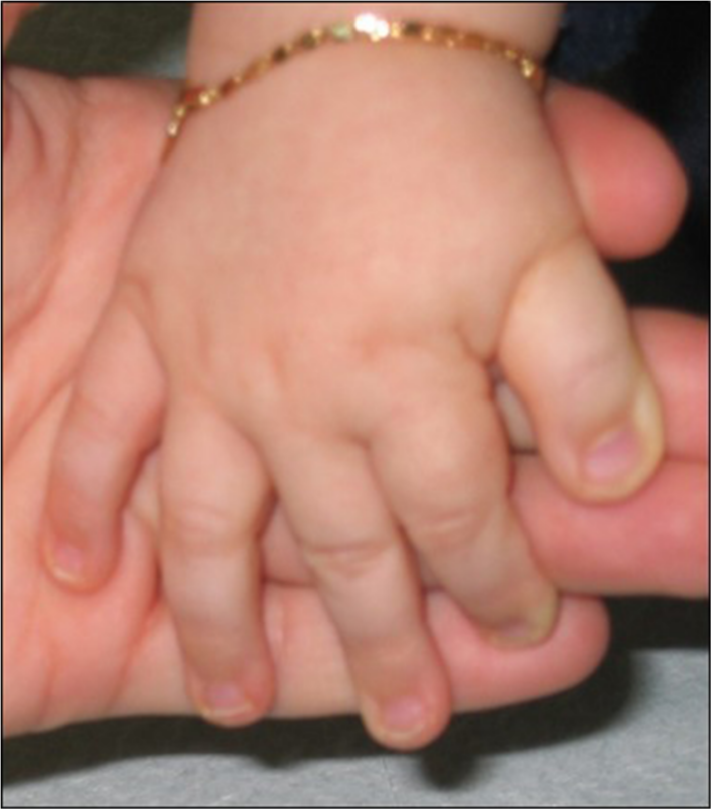
**Typical hands of a RSTS patient, including enlarged first finger and clinodactyly of the fifth finger.**

RSTS is characterized by slow development of height and weight, microcephaly, dysmorphic facial features, broad thumbs, and big toes [18]. The prenatal development is normal, with average or near-normal growth parameters at birth. The growth charts typically approach the lower limits of normality in the first postnatal period, primarily reflecting hypo-feeding exacerbated by gastro-esophageal reflux. Subsequently, the tendency of overweight or obesity (earlier in males than females) can be observed during adolescence. Specific and recently reviewed growth charts are essential for appropriate assessment of the growth of affected individuals [18]. Facial features are primarily characterized by low frontal hairline, arched/thick eyebrows, downslanting of palpebral fissures, a protruding beaked nose with columella below alae nasi, dysplastic and low-set ears, an arched palate, mild micrognathia, dental anomalies (altered conformation, malocclusion, and overcrowding of teeth), and atypical smile (“grimacing”) with nearly completely closed eyes (Figure 1). The feet and hands typically present an enlarged first finger and clinodactyly of the fifth finger (Figure 2), whereas polydactyly with bifid thumbs and first toes is rarely observed. Other skeletal anomalies include abducted thumbs, vertebral anomalies, ligamentous laxity, severe and prolonged aseptic inflammation of the femur head, anomalies similar with Perthes disease (3%), and occasionally slipped capital femoral epiphysis [19,20]. Particularly, high risk of cervical vertebral abnormalities (instability of C1–C2, os odontoideum, hypoplasia of the dens, fusion of the cervical vertebrae) has been reported [21-23], with possible stenosis at the craniovertebral junction, which may cause cervical myelopathy. Complex neuroradiological issues including corpus callosum dysgenesis (17%) [24,25], Chiari type I malformation with or without syringomyelia [25-28], Dandy Walker malformation and hydrocephalus [29,30], and tethered cord [27,31] have been reported and are still under investigation. Cerebrovascular abnormalities such as spontaneous dissection of the supraaortic arteries [32] and cerebral infarction due to dissecting aneurysm of the anterior cerebral artery have also been reported [33]. However, any organ can be affected in RSTS patients. Possible malformations, medical problems, and complications include (Table 1):

- conductive and/or sensorineural deafness, recurrent middle ear infections, recurrent respiratory infections, immune deficiencies [34-36];
- nonspecific abnormalities of electroencephalography (EEG) (57-66%) and seizures (25%) [37,38];
- cataract, unilateral or bilateral iris/retinal/optic nerve coloboma (9-11%), glaucoma, lacrimal duct obstructions (38-47%), refractive errors (41-56%), and strabismus (60-71%) [39-41]. In addition, Jacobs *et al*. described for the first time peripheral avascularity with fluorescein angiography in 2012 [42];
- dental problems: talon cusps (73%), enamel hypoplasia, and abnormal tooth number [43,44];
- congenital heart diseases: atrial septal defect, ventricular septal defect, patent ductus arteriosus, coarctation of the aorta, pulmonic stenosis, bicuspid aortic valve, pseudotruncus, aortic stenosis, dextrocardia, vascular rings, and conduction disorders (24-38%) [45]. Occasional association of hypoplastic left heart with RSTS has also been reported [46];
- renal malformations (52%) and cryptorchidism (78-100%) [47];
- endocrine disorders: congenital hypothyroidism [48,49], thyroid hypoplasia, GH deficiency, and pituitary hypoplasia [28];
- gastrointestinal disorders: gastroesophageal reflux, constipation (40-74%), and megacolon/Hirschsprung disease [47,50];
- obstructive sleep apnea, anesthetic and intubation complications [51,52];
- skin problems including pilomatrixomas, ingrown toenails, paronychia, and the tendency to form keloids (24%) [53,54];
- cancers, particularly of neural and developmental origins (neuroblastoma, medulloblastoma, oligodendroglioma, meningeoma, pheochromocytoma, rhabdomyosarcoma, leiomyosarcoma, seminoma, odontoma, choristoma, and pilomatrixomas [55-63]. Leukemia and lymphoma have also been reported [55];
- hirsutism

**Table 1:** **The incidence of a number of typical features of RSTS**

**Feature**	**Incidence (%)**
Typical facial features	100
Intellectual disability	~100
Cryptorchidism	78-100
Microcephaly	35-94
Broad thumbs/halluces	96
Speech delay	90
Recurrent respiratory infections	75
Delayed bone age	74
Constipation	40-74
Talon cusps	73
Gastroesophageal reflux	68
EEG abnormalities	57-66
Renal anomalies	52
Refractive defects, glaucoma, retinopathy	>50
Congenital heart defects	24-38
Seizures	25
Keloids	24
Deafness	24
Growth retardation	21
Malignant tumors	3-10
Spinal cord tethering	<5

.

The neonatal period of individuals with RSTS is typically characterized by hypotonia and delayed psycho-motor development, with variable degrees of intellectual disability. For example, the intelligence quotient (IQ) score of RSTS patients in neonatal period usually ranges from 25 to 79 (average: 36–51) [64,65]. In 2009, Galèra *et al*. described three RSTS cardinal features, short attention span, motor stereotypies, and poor coordination [66]. Both RSTS patients with classical RSTS and mild intellectual disability [64,67] and RSTS patients with atypical RSTS and mild intellectual disability have been reported [68]. Therefore, in the mildest cases, an early diagnosis is particularly difficult, and the main stages of development must be strictly followed to rapidly initiate specific and individualized stimulation. In addition, although RSTS patients usually have friendly and sociable characteristics, behavioral disorders, mood swings, and obsessive-compulsive disorders can still be observed, particularly in adulthood [64,65,69].

### Transition and healthcare in adulthood

Over 90% individuals with RSTS survive to adulthood [70], and healthcare for these patients is particularly complex, time-consuming, and often not standardized in specific guidelines. The medical problems of most genetic syndromes often change with ages and there is limited knowledge about the management of adults with genetic syndromes [71]. Adult individuals with RSTS have been documented [13,59,68,72], but only a few review studies on adults with RSTS are available [40,57,69,73]. In these review studies, adult RSTS patients had relevant medical problems and most of them had overweight or obesity. A number of behavioural phenotypes such as anxiety, mood instability, and aggressive behaviour can appear during adolescence. Caregivers reported decreased abilities over time in 32% RSTS subjects and some worsening behaviors in 37% RSTS patients, which is consistent with the report by Hennekam *et al*. in 1992 [64]. Therefore, follow-up care is important to identify and treat the psychiatric problems that emerge with age [65]. Finally, the prevalence of RSTS may be higher than original estimation due to late diagnosis particularly in the milder cases [74].

### Diagnostic approaches

Individuals with suspected RSTS should be evaluated by pediatric geneticists knowledgeable in dysmorphology. A number of molecular techniques are widely used in genetic analyses of RSTS. Among the assays, karyotype analysis may show rare cytogenetically visible abnormalities (translocations, inversions, or deletions); although the result is usually normal, this assessment should in any case be performed to identify possible rearrangements. FISH can identify microdeletions, with a detection rate of 5-10% [38,75]. Deletion/duplication analysis testing identifies exonic or whole-gene deletions/duplications not detectable by sequence analysis of the coding and flanking intronic regions of genomic DNA. Various methods may be used (quantitative PCR, long-range PCR, multiplex ligation-dependent probe amplification (MLPA), and chromosomal microarray). Stef *et al.* detected deletions in 17 (20.5%) of 83 patients using array-CGH and quantitative multiplex fluorescent-PCR [76]. Molecular analysis can also identify mutations in *CREBBP* and *EP300* genes. Pathogenic variants of the *CREBBP* gene were identified in 50-70% of RSTS individuals [8,9,38,77], while mutations in the *EP300* gene have been reported in about 5-8% RSTS patients by Roelfsema et al. [2005], Bartholdi et al. [2007], Negri et al. [2014] [11,12,16].

### Genotype-phenotype correlations

Little is known about genotype-phenotype correlations of RSTS. A severe phenotype has been reported in RSTS patients with large deletions [78], but other studies [76,79] do not support this genotype-phenotype association. However, an association between lower IQ and autistic features with large deletions in RSTS patients is possible [38]. Therefore, Calì *et al*. recommended MLPA that can identify these large deletions for screening RSTS patients with lower IQ and autistic features [80]. Mutations outside the histone acetyltransferase (HAT) domain were associated with a mild phenotype [Spena *et al*., submitted]. In addition, somatic mosaicism may also be associated with mild RSTS [9,81,82]. Less than 20 RSTS patients with *EP300* mutations have been identified and characterized till now. *EP300* mutations have been associated with preeclampsia in women carrying a pregnancy affected by RSTS; skin involvement and a mild phenotype in skeletal abnormalities and neuropsychiatric issues are described [12-16,83].

### Genetic counseling

Most RSTS cases are sporadic and only a few RSTS cases affecting siblings have been reported to date [1,82,84-86]. Vertical transmission is extremely rare [75,86-89]. While the recurrence risk of RSTS is generally low, proper genetic counseling should be provided for prenatal diagnosis of RSTS. Somatic mosacism, for example, was confirmed in the clinically unaffected father of a boy with RSTS [82] and in the mildly affected father of three females with RSTS [89]. In addition, germline mosaicism was hypothesized in two RSTS cases [17,82]. Based on these reports, the recurrence risk of RSTS is approximately 0.5-1%.

### Management

While significant advance in the knowledge of clinical manifestations and natural history of RSTS has been made, guidelines for the healthcare and follow-up care of RSTS have not been well updated after the proposal of Wiley et al. in 2003 (Table 2) [37]. Novel genetic and epigenetic therapies may be promising approaches for the treatment of RSTS [90,91], but there is an urgent need to improve and personalize the standard follow up protocol. On the basis of our knowledge and of the critical aspects that we discuss below, we drafted our proposal for follow-up (Table 3).

**Table 2:** **Traditional medical guidelines for RSTS management**

	**Diagnosis**	**6 M**	**1Y**	**18 M**	**2Y**	**30 M**	**>3Y (yearly)**
Audiologic evaluation	X	X	X	X	X	X	X
Ophtalmologic evaluation	X	X		X		X	X
Orthopedic evaluation	X	X	X	X	X	X	X
Cardiologic evaluation*	X						
Pressure measurement							X
Renal US scan*	X						
Odonthoiatric evaluation			X	X	X	X	X
Genetic counseling	X						

M = months, Y = years.

*Follow-up if necessary.

**Table 3:** **Our proposal for medical guidelines in patients with RSTS**

	**Diagnosis**	**6 M**	**1Y**	**18 M**	**2Y**	**30 M**	**>3Y (yearly)**	**Adolescent age**
Brain and medullary NMR*	X							X
Neuropsychiatric evaluation	X							X
Audiologic evaluation	X	X	X	X	X	X	X	X
Ophtalmologic evaluation	X	X		X		X	X	X
Orthopedic evaluation	X	X	X	X	X	X	X	X
Cardiologic evaluation*	X							X
Pressure measurement							X	X
Renal US scan*	X							X
Odonthoiatric evaluation			X	X	X	X	X	X
Endocrinological evaluation*	X					X		X
Dermatologic evaluation*	X							X
Genetic counseling	X							X

M = months, Y = years.

*Follow-up if necessary.

Management should be adjusted in adolescent age, for the known differences in some issues (ophthalmological features, tendency to obesity and mood disorders in particular).

### Unknown and critical issues in RSTS

Substantial progress has been made in studies of the genetic basis and medical issues of RSTS, which contributes to initial clinical diagnosis and subsequent confirmation through molecular analyses. Given the complexity and rarity of this syndrome, there are still numerous unanswered questions about RSTS. Therefore, further investigations should be focused. on clinical diagnosis and management as well as on genotype-phenotype correlation.

Based on our experience, abnormal growth patterns as seen on standard growth charts should be highlighted in the diagnostic criteria of RSTS. In addition, clinical diagnostic criteria and screening could be further classified according to prenatal, childhood, and adolescent periods. Particularly, the presence of normal growth *in utero*, associated with other markers such as broad thumbs/halluces and other malformations, is useful in differential diagnosis of RSTS from other syndromes (i.e., Cornelia de Lange syndrome). Pediatric geneticists should pay more attention to widened distal phalanges: reviewing patients’ photos sent to molecular analyses we found in a great majority this sign, not reported in clinical charts. Enlarged first finger is a feature largely known, but also common in other syndromes such as acrocephalopolysyndactyly, whereas distal phalanges conformation seems more specific for RSTS. Moreover, talon cusps that are often overlooked are also highly specific for RSTS. A multicenter prevalence study of brain and spine abnormalities is needed, in view of the several reports and of the both diagnostic and prognostic meaning of these features; a screening brain/medullary MRI could be useful in addition to basic diagnostic work-up. Furthermore, regarding endocrinological features, more informations were recently added, regarding in particular thyroid shape and function, and we are personally aware of other two cases with mild hypothyiroidism and small thyroid. In addition to intellectual disability, some behavioral changes are known for RSTS patients, but no significant evidence supports the diagnostic values of these neuropsychiatric features in RSTS diagnosis. Therefore, no neuropsychiatric features are strong enough to be included in the diagnostic criteria of RSTS although some features may be more suggestive of *EP300* mutations. In these cases, ID is mild or absent, and behavioral disturbance (i.e. anxiety) predominates. Other features suggestive of *EP300* mutations include pre-eclampsia [92] and less significant abnormalities in the firs digit, giving the cue for drawing up differential criteria for *EP300*, and for a more precise and individualized laboratory flow-chart. To the best of our knowledge, this is the only possible change in the order of molecular investigations, as other genotype-phenotype correlations are only tentative. Numerous non-specific complications may occur during the follow-up care of RSTS; therefore, it is difficult to establish a general and efficient follow-up protocol. In addition, no robust genotype-phenotype correlations have been identified. In general, orthopaedic follow-up, dietary monitoring in adolescence and neuropsychiatric periods, and ophthalmologic evaluations in adults should be focused. A less strict follow-up protocol may be only appropriate for RSTS patients with *EP300* mutations [16], with focus on skin problems (pilomatrixomas and nevi) that are likely more frequent than in patients with *CREBBP* mutations. Regarding genetic counselling, salivary brush and genetic tests are important for the evaluation of recurrence risk in parents with germinal and somatic mosaicism.

A discussion about critical aspects of progresses in understanding of RSTS etiopathogenesis is out of the scope of this review, but different mouse models have been made with interesting results [90].

## Conclusions

RSTS is an extremely rare condition for which some clinical aspects have been clearly identified, but a lot of studies are ongoing and needed. Multicenter studies are needed to expand our knowledge on the clinical phenotype, identify specific genotype-phenotype correlations, evaluate the presence of somatic mosaicisms to better define mild phenotypes, and identify new candidate genes. The ultimate goal of these studies is to extend our current knowledge concerning this syndrome and to define new international guidelines for diagnosis, care and treatment of patients with RSTS.

## Summary

RSTS is an extremely rare multiple congenital anomaly/intellectual disability syndrome, with an estimated prevalence of one case per 125,000 live births. No precise diagnostic criteria have been defined, although the distinctive features include typical facial features, microcephaly, broad thumbs and first toes, intellectual disability and postnatal growth retardation. RSTS is mainly characterized by poor growth in height and weight, microcephaly, dysmorphic facial features, broad thumbs and big toe. Several organs and systems may be affected, but none of other signs or symptoms can be considered pathognomonic. More than 90% of individuals with disabilities survive into adulthood, and health care for these patients is particularly complex, time-consuming, often not standardized in specific guidelines. The gene most frequently involved is cyclic-AMP-regulated enhancer binding protein (*CREBBP*); alterations in the E1A-binding protein p300 (*EP300*) have also been detected, but many cases have only been clinically diagnosed. Future multicenter studies are necessary to expand our knowledge on the clinical phenotype, identify specific genotype-phenotype correlations, evaluate the presence of somatic mosaicisms to better define mild phenotypes, and identify new candidate genes. The ultimate goal of these studies is to extend our current knowledge concerning this syndrome and to define new international guidelines for diagnosis, care and treatment of patients with RSTS.

### Ethical approval

The follow-up studies of RSTS children performed by the authors have been approved by the Ethics Committee of Fondazione IRCCS Ca’ Granda Ospedale Maggiore Policlinico, Milan, Italy.

### Consent

Written informed consent was obtained from the patients’ parents for the publication of this report and any accompanying images.

## Abbreviations

RSTS: Rubinstein-Taybi syndrome
*CREBBP*: Cyclic-AMP-regulated enhancer binding protein
*EP300*: E1A binding protein p300
FISH: Fluorescent *in situ* hybridization
HAT: Histone acetyltransferase
IQ: Intelligence quotient
MLPA: Multiplex ligation-dependent probe amplification
